# Detection of Common Bile Duct Stones in Mild Acute Biliary Pancreatitis Using Magnetic Resonance Cholangiopancreatography

**DOI:** 10.1155/2018/5216089

**Published:** 2018-10-22

**Authors:** David Aranovich, Veacheslav Zilbermints, Natalia Goldberg, Oleg Kaminsky

**Affiliations:** ^1^Department of General Surgery, Beilinson Hospital, Rabin Medical Center, Petach-Tikva, Israel; ^2^Affiliated with Sackler Medical School, Tel-Aviv University, Tel-Aviv, Israel; ^3^Department of Diagnostic Radiology, Beilinson Hospital, Rabin Medical Center, Petach-Tikva, Israel

## Abstract

**Background:**

All patients with mild acute biliary pancreatitis should undergo early cholecystectomy. Whether routine common bile duct (CBD) imaging should be employed before the surgical procedure in these patients is a matter of current controversy. The aim of this study was to investigate the rate of detection of CBD stones using magnetic resonance cholangiopancreatography (MRCP) at different time intervals from admission.

**Methods:**

From January 1, 2011, through December 31, 2016, 72 patients with acute biliary pancreatitis underwent MRCP. Fifty-six (*n*=56) of them with mild biliary pancreatitis met the study criteria. The patients were divided into two groups. Group A did not have stones in the CBD (*n*=45), and Group B had stones in the CBD (*n*=11). The time from admission to MRCP was divided into several periods (day 1 through day 180), and the presence of the CBD stones on MRCP was weighted against remoteness from admission. Liver chemistry profiles were compared between the groups on admission and before the MRCP.

**Results:**

The cumulative rate of choledocholithiasis was 19.7% (Group B, *n*=11). Forty-five patients (Group A, *n*=45, 80.3%) did not have gallstones in the CBD. Eight patients with choledocholithiasis (8/56, 14.2%) were detected during the first 10 days from admission out of 27 patients. In patients who underwent MRCP between days 11 and 20, choledocholithiasis was found in two patients (2/56, 3.5%) and in one patient between days 21 and 30 (1/56, 1.8%). No stones were found in patients who underwent MRCP beyond 30 days from admission. Liver chemistry profiles did not show a significant difference in both groups. CBD dilatation was observed at presentation in 11 patients (*n*=11/56), 6 in Group A (6/45, 13.3%) and 5 in Group B (5/11, 45.5%) (*p*=0.016).

**Conclusions:**

Routine CBD evaluation should be encouraged after mild acute biliary pancreatitis. Early performance of MRCP gives high yield in selecting the patients for endoscopic retrograde cholangiopancreatography (ERCP) before cholecystectomy. A liver chemistry profile either on admission or before MRCP cannot predict the presence of CBD stones.

## 1. Introduction

The passage of gallstones from the gallbladder through the common bile duct (CBD) into the duodenum has been implicated in the pathophysiology of acute biliary pancreatitis [1]. Most patients with acute biliary pancreatitis experience a rather mild course of the disease, with typical abdominal pain and transient elevation in liver function test (LFT) results and pancreatic enzyme levels [2]. Knowledge on the common bile duct (CBD) clearance in any symptomatic gallstone-related condition is anticipated before removal of the gallbladder. However, the event of stone passage and spontaneous CBD clearance is uncertain, even following complete clinical recovery and biochemical resolution of acute biliary pancreatitis. Our policy is to perform CBD evaluation with magnetic resonance cholangiopancreatography (MRCP) or intraoperative cholangiography (IOC) before elective cholecystectomy for acute biliary pancreatitis. MRCP is commonly performed either during index hospitalization or on an outpatient basis, depending on the availability of the MRCP.

The same admission cholecystectomy is recommended in patients with mild biliary pancreatitis, which shows satisfactory recovery with conservative treatment. Nevertheless, the same admission surgery is not always implemented because of operating room availability or patients' preferences. Thus, many patients are discharged home upon recovery and scheduled for elective procedures later on. This less favorable situation creates a subset of patients recovered from an initial episode of mild biliary pancreatitis on the one hand, but with an uncertain risk of choledocholithiasis on the other hand. We prefer to screen these patients with MRCP for occult choledocholithiasis prior to cholecystectomy at any time interval and irrespective of the liver chemistry profile.

The purpose of this study was to evaluate the rate of choledocholithiasis detection using MRCP, after mild biliary pancreatitis, across different time intervals after index admission and to compare liver chemistry profiles on admission and before MRCP in patients with and without choledocholithiasis.

## 2. Methods

The study was approved by the Institutional Review Board of the Rabin Medical Center, protocol number 0731-16-RMC. Medical records were extracted from the hospital's electronic database system.

Patients with acute biliary pancreatitis were identified using the ICD-9 code. The procedure code for MRCP was combined with the ICD code for acute pancreatitis in the computer search algorithm to select those patients who underwent MRCP prior to cholecystectomy.

From January 1, 2011, through December 31, 2016, 72 patients with acute biliary pancreatitis who underwent preoperative CBD evaluation using MRCP were identified. The diagnosis of acute biliary pancreatitis was based on clinical grounds, namely, upper abdominal pain, abdominal tenderness, and elevated levels of bilirubin, transaminases (alanine aminotransferase (ALT) and aspartate aminotransferase (AST)), alkaline phosphatase (ALKP), amylase, lipase, and the visualization of gallstones using abdominal ultrasonography [3]. Patients with mild acute biliary pancreatitis were selected from the records by the absence of organ failure and local or systemic complications and a Ranson's score of less than three [4]. Patients with severe pancreatitis, one or more organ failures, or documented pancreatic necrosis were excluded from the study (*n*=16).

Based on these criteria, we selected for retrospective analysis 56 patients with mild acute biliary pancreatitis, who underwent MRCP before planned cholecystectomy. All patients made an uneventful recovery and were scheduled for cholecystectomy. The patients selected for the study were asymptomatic between the episode of mild biliary pancreatitis and the cholecystectomy. Laparoscopic cholecystectomy was performed in all cases without intraoperative cholangiography.

The patients were divided into two groups based on the MRCP findings. Group A patients did not have stones in the CBD (*n*=45), while Group B patients had stones on MRCP and subsequently underwent endoscopic retrograde cholangiopancreatography (ERCP) before cholecystectomy (*n*=11). Relevant data relating to demography, liver chemistry profiles and pancreatic enzyme levels on admission and prior to MRCP, time from admission to MRCP, and CBD dilatation on admission ultrasonography were analyzed. The time from admission until MRCP was divided into several periods (day 1 through day 180), and the presence of the CBD stones on MRCP was weighed against remoteness from the index admission. Laboratory and demographic variables are presented as mean ± SD. The variables in the two groups were compared using the two-tailed Student's *t* test, chi-square test, and Mann–Whitney U test, as appropriate. A *p* value less than 0.05 was accepted as a criterion for statistical significance.

## 3. Results

Thirty-six out of 56 patients (36/56, 64%) were female. The median age of the patients was 62 years (range, 28–89). The median hospital stay length was 9 days (range, 2–17). Based on the CBD evaluation on MRCP, 45 patients (Group A, *n*=45, 80.3%) did not have gallstones in the CBD, while 11 patients (Group B, *n*=11, 19.7%) were found to have choledocholithiasis, after an episode of mild acute biliary pancreatitis. There were no significant differences in the two groups with respect to sex ratio (*p*=0.1), age (*p*=0.4), and major comorbidities.

The demographic data and biochemical parameters at presentation and before MRCP in both groups are summarized in Table 1.

**Figure 1:**
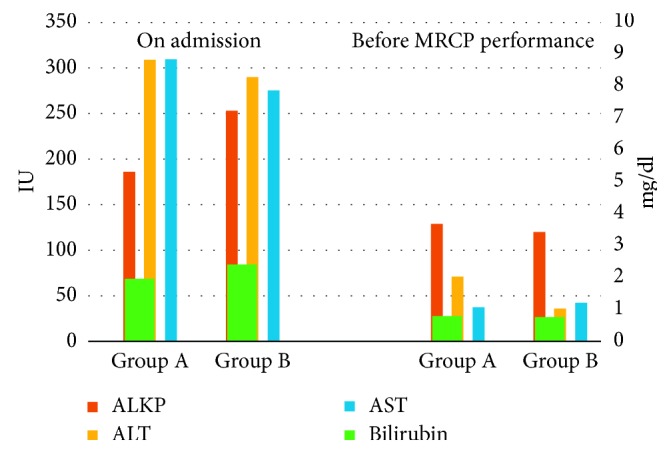
Comparison of liver chemistry profiles on admission and before MRCP. Primary axis (right): international units (IU) for ALKP and transaminases (AST and ALT); secondary axis (left): mg/dl for bilirubin.

As shown in the table, no significant disparities between the groups were observed in laboratory parameters on admission and before MRCP, such as total bilirubin, ALKP, ALT, AST, amylase, and lipase. Liver chemistry profiles in both groups on admission and before MRCP are graphically presented in Figure 1.

CBD dilatation was observed at presentation on abdominal ultrasonography in 11 patients (*n*=11/56), 6 of them in Group A (6/45, 13.3%) and 5 in Group B (5/11, 45.5%) (*p*=0.016).

Overall, 42 patients out of a total of 56 (42/56) underwent MRCP within 30 days from admission. All MRCP-positive patients with choledocholithiasis were detected within this time frame (11/42).

The cumulative rate of choledocholithiasis was 19.7% (Group B, *n*=11). Forty-five patients (Group A, *n*=45, 80.3%) did not have gallstones in the CBD.

In 27 patients (27/56), MRCP was performed within 10 days from admission. Of these 27 patients, the CBD stones were found in 8, which comprises 14.2% (8/56) of all study population. In the patients who underwent MRCP between days 11 and 20, stones were found in 2 out of 10 patients (2/10), which comprises 3.5% (2/56) of all patients, while in 1 additional patient out of 5 (1/5) who underwent MRCP between days 21 and 30, which comprises 1.8% of all patients in the study. In 14 patients, MRCP was performed beyond 30 days from admission, and no stones were found during this period.

**Figure 2:**
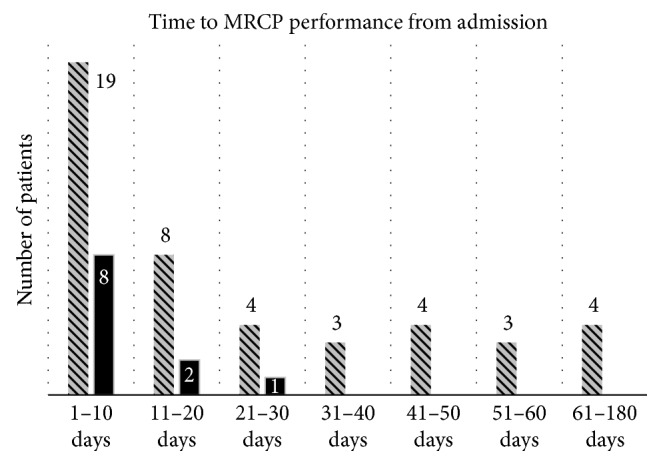
Rate of CBD stone detection across different time intervals after admission. Pattern-filled bars correspond to patients without MRCP-detected CBD stones. Solid bars correspond to patients with MRCP-detected CBD stones. Corresponding numbers of the patients are indicated.

Figure 2 depicts the detection rate of MRCP-detected CBD stones across different time intervals, as described above.

## 4. Discussion

Current guidelines recommend early cholecystectomy in patients recovering from an index episode of mild acute biliary pancreatitis. This recommendation is based on studies that consistently show the benefit of early surgery in terms of lowering hospitalization costs [5–10] and lowering the recurrence of CBD stone-related issues and readmissions, without compromising safety of the laparoscopic procedure and conversion rates [6, 11, 12]. While there is a common agreement that ERCP should be performed early in pancreatitis with concomitant cholangitis and/or CBD obstruction [8, 13, 14], few areas of uncertainty exist regarding CBD evaluation in the subgroup of patients with improving clinical condition and normal LFTs.

These patients may benefit from other CBD imaging techniques, namely, IOC and MRCP. According to the UK guidelines for the management of acute pancreatitis (Working Party of the British Society of Gastroenterology), imaging of the CBD with operative cholangiography should be done in every patient with gallstones and pancreatitis [15]. These recommendations are consonant with the American College of Gastroenterology guidelines, which also advocate for laparoscopic cholecystectomy with intraoperative cholangiography followed by operative or postoperative ERCP, should CBD stones be discovered during the procedure [16].

On the contrary, it is well known that the majority of patients with acute biliary pancreatitis will pass the stones spontaneously. Tranter and Thompson found that up to 80% of patients in the history of acute biliary pancreatitis had passed their stones before planned cholecystectomy [17]. Based on this knowledge, some groups proposed a selective approach to IOC and complete omission of CBD evaluation in patients with a presumed minimal risk of retained stones after acute biliary pancreatitis [18, 19].

Very few studies have addressed the level of spontaneous passage of CBD stones in acute biliary pancreatitis across different time intervals. Our results are concordant with those reported in the literature. Cavdar et al. followed up 60 patients with acute biliary pancreatitis who underwent MRCP at different time intervals. In their series, 33% of patients had cholelithiasis during the first 4 days, and 80% of them had stones initially. The authors concluded that MRCP performed in the second week of acute biliary pancreatitis showed higher efficacy in selecting patients for ERCP prior to surgery [20].

We found that 14.2% (8/56) of stones were detected during the first 10 days after admission for mild acute biliary pancreatitis. During the next 10 days, 3.5% (2/56) of CBD stones were detected and 1.8% (1/56) within the following 10 days. No cases of choledocholithiasis were found beyond day 30 in the remaining 14 patients.

There were no obvious differences in the biochemical profile between the study groups. CBD dilatation was observed in 45.5% of patients with choledocholithiasis (Group B) and in only 13.3% without choledocholithiasis (Group A). While CBD dilatation observed through admission ultrasonography was the only statistically significant predictor of CBD stones on MRCP (*p*=0.016), a substantial number of dilated CBDs were also found in the stone-negative group. Thus, we cannot recommend CBD dilatation as a sole criterion for the selection of patients for preoperative investigation.

It is evident that the majority of our patients underwent MRCP either during the index admission or shortly thereafter. In general, MRCP scheduling for mild acute biliary pancreatitis is based on availability in our institution. The retrospective nature of the study may introduce selection bias, such as favoring of patients with higher probability of choledocholithiasis to have MRCP earlier in the course of their disease and delaying investigations in poor surgical candidates. However, we did not find noticeable differences in indications for the timing to perform MRCP. Our study also reports a less favorable scenario, when some patients were operated on with significant delay. Although no CBD stones were found beyond the 30-day period, but in lieu of a small number of patients in this subgroup, we should be cautious in interpretation of these data.

Therefore, we conclude that the majority of the common bile duct stones (14.2%) are found within 10 days from the index admission for mild acute biliary pancreatitis. This time frame corresponds to many international guidelines advocating for the same admission cholecystectomy in patients with mild acute biliary pancreatitis. Thus, routine CBD evaluation should be encouraged after mild acute biliary pancreatitis, regardless of the liver chemistry profile, and preoperative MRCP will ensure the highest yield in detecting choledocholithiasis before the planned surgical procedure.

## Figures and Tables

**Table 1:** Patient characteristics in two study groups on admission and before MRCP.

Characteristics	Unit	MRCP negative (*n*=45)	MRCP positive (*n*=11)		MRCP negative (*n*=45)	MRCP positive (*n*=11)	
		Group A	Group B	*p* value	Group A	Group B	*p* value
		On admission			Before MRCP		
Male/female	*n*	15/30	5/6	0.1			
Age	yrs	**63** (25–89) ± 21.5	**57** (28–89) ± 22	0.4			
Total bilirubin	mg/dl	**1.96** (0.2–6.58) ± 1.58	**2.4** (1.1–5.7) ± 1.6	0.4	**0.8** (0.3–1.91) ± 0.4	**0.78** (0.4–2.7) ± 0.67	0.9
ALKP	IU/dl	**186** (56–422) ± 97	**254** (112–470) ±117	0.04	**129** (54–422) ± 82	**120** (62–198) ± 43	0.7
ALT	IU/dl	**309** (14–972) ± 282	**298** (151–521) ±123	0.4	**71** (8–359) ± 91	**36** (12–135) ± 36	0.4
AST	IU/dl	**310** (15–1384) ± 299	**276** (87–659) ± 181	0.7	**38** (14–136) ± 34	**43** (14–177) ± 54	0.6
Amylase	IU/dl	**1222** (101–6348) ± 1291	**1612** (54–4077) ± 1492	0.3	**98** (40–517) ± 102	**83** (47–119) ± 29	0.7
Lipase	IU/dl	**2980** (45–18250) ± 3736	**2048** (122–7120) ± 2351	0.4	**75** (11–700) ± 140	**75** (48–103) ± 39	0.9
Dilated CBD	*n*	6/45	5/11	0.016			

Results are presented as mean ± SD. AST: aspartate aminotransferase; ALT: alanine aminotransferase; ALKP: alkaline phosphatase; CBD: common bile duct.

## Data Availability

The data used to support the findings of this study are available from the corresponding author upon request.
